# Real-Time, Label-Free Detection of Biomolecular Interactions in Sandwich Assays by the Oblique-Incidence Reflectivity Difference Technique

**DOI:** 10.3390/s141223307

**Published:** 2014-12-05

**Authors:** Yung-Shin Sun, Xiangdong Zhu

**Affiliations:** 1 Department of Physics, Fu-Jen Catholic University, New Taipei City 24205, Taiwan; 2 Department of Physics, University of California at Davis, Davis, CA 95616, USA; E-Mail: xdzhu@physics.ucdavis.edu

**Keywords:** label-free biosensor, oblique-incidence reflectivity difference (OI-RD), protein-protein interaction, protein-small molecule interaction, real-time kinetics

## Abstract

One of the most important goals in proteomics is to detect the real-time kinetics of diverse biomolecular interactions. Fluorescence, which requires extrinsic tags, is a commonly and widely used method because of its high convenience and sensitivity. However, in order to maintain the conformational and functional integrality of biomolecules, label-free detection methods are highly under demand. We have developed the oblique-incidence reflectivity difference (OI-RD) technique for label-free, kinetic measurements of protein-biomolecule interactions. Incorporating the total internal refection geometry into the OI-RD technique, we are able to detect as low as 0.1% of a protein monolayer, and this sensitivity is comparable with other label-free techniques such as surface plasmon resonance (SPR). The unique advantage of OI-RD over SPR is no need for dielectric layers. Moreover, using a photodiode array as the detector enables multi-channel detection and also eliminates the over-time signal drift. In this paper, we demonstrate the applicability and feasibility of the OI-RD technique by measuring the kinetics of protein-protein and protein-small molecule interactions in sandwich assays.

## 1. Introduction

In modern proteomics, it is important and necessary to be able to analyze the interactions between proteins and other biomolecules. Quantitative analysis of binding kinetics helps biologists to better understand the structures and functions of these proteins. Traditional methods such as chromatography and immunoprecipitation are widely used in detecting protein-protein interactions, but they are limited to qualitative measurements. Recently, various real-time techniques with the capacity of obtaining binding affinities have been developed, and some of them have become commercially available. Most of these real-time methods are based on specifically (fluorescence-, enzyme-, radioisotope-) labeled compounds. For example, fluorescence-labeling is widely used for detecting biomolecular interactions in both real-time kinetics [1,2] and the so-called “end-point” microarray format [3]. Usually, one of the reacting pair is tagged chemically with fluorescent molecules (Cy3-, Cy5-dye or quantum-dot). These fluorophores provide very high sensitivities, and with sophisticated scanning and detecting instruments, high throughput and wide dynamic range are available. However, some issues regarding to these “labeled” methods have been pointed out [4–6]. First, labeling proteins could change their conformational and functional properties, especially for those which are very small. These extrinsic tags can have significant influences on the binding affinities and isothermal equilibrium of these proteins. Second, the heterogeneity of proteins makes quantitative analysis difficult because the efficiency of labeling may vary from protein to protein. Thirdly, labeling proteins can be very laborious as well as expensive work. As a result, label-free, real-time detections that can circumvent these problems are highly necessary.

Some label-free methods based on light interference and ellipsometry were developed and tested for detecting biomolecular reactions early in the 1940s [7]. Recently, more and more modern optical techniques have been applied in designing label-free biosensors for applications not only in the area of biological research, but also in clinical diagnostics and environmental control [8]. Surface plasmon resonance (SPR) works by measuring the changes in coupling conditions (wavelength of incident light and/or resonant angle) in response to changes in refractive index of surface-bound molecules [9–11]. Resonant mirror (RM) is similar to SPR in construction and working principles, but the dielectric materials used in the resonant layer (typically Ag or Au in SPR) can give much lower losses and hence allow for longer interaction lengths [12–14]. Reflectometric interference spectroscopy (RIfS) determines the changes in thickness and refractive index of a thin deposited layer by measuring the interference of light source caused by partial reflection at the interface [15–18]. All these methods are applicable in kinetic analysis of biomolecular interactions and are also of very high sensitivity. For example, the detection limits (or sensitivities) are about 0.78, 7.36, and 1.50 pg protein/mm^2^ for SPR, RM, and RIfS, respectively [19].

In this study, we report using the oblique-incidence reflectivity difference (OI-RD) technique for real-time, label-free detection of biomolecular interactions in sandwich assays. The OI-RD microscopy, a particular and the most sensitive form of optical elliposometry, has a comparable sensitivity (few pg protein/mm^2^) to all above-mentioned methods. This technique is suitable for both real-time and end-point measurements, and, comparing to SPR or RM, no dielectric layers is required. In the past few years, we have applied OI-RD microscopes in various detections such as optical properties of thin films (gas or solid phase) upon substrates [20–22] and biomolecular interactions in microarray format [23–27]. Using the OI-RD microscopy and the sandwich assays, real-time curves of antibody binding to surface-immobilized antigen and antigen binding to surface-immobilized antibody were monitored. Experimental results indicated that these two assays gave different binding signals, which might due to surface heterogeneity of immobilized antibodies and antigens. Also, the bindings in a sandwich assay of biotin-streptavidin-biotin conjugated bovine serum albumin (BSA) were monitored using an OI-RD microscope. Being able to study the heterogeneity of proteins and detect small molecular protein ligands, the feasibility of the OI-RD technique, in combination with microarrays, can definitely benefit the development of proteomics, or the large-scale studies of proteins, particularly their structures and functions.

## 2. Experimental Section

### 2.1. The OI-RD Technique

The working principles of the OI-RD technique were detailed in our previous works [24,28]. OI-RD is a particular form of optical ellipsometry which measures the differential changes in both phase and magnitude of the reflectivities for *p*- and *s*-polarized components of a monochromatic light in response to small changes in physical and chemical properties of deposited thin biomolecular layers. When the layer thickness *d* is much less than the optical wavelength *λ*, the reflectivity difference can be express as:

(1) $$\Delta_{p}-\Delta_{s}≅i\alpha \frac{\left({ɛ}_{d}-{ɛ}_{0})({ɛ}_{d}-{ɛ}_{s}\right)}{{ɛ}_{d}}\left(\frac{d}{\lambda }\right)$$

where:

(2) $$\alpha \equiv -\left[\frac{{4\pi {ɛ}_{0}}^{1/2}\cos\theta }{\left({ɛ}_{0}-{ɛ}_{s})(\cot^{2}\theta -{ɛ}_{s}/{ɛ}_{0}\right)}\right]$$

In these equations, Δ*_p_* ≡ (*r_p_*−*r_p_*_0_)/*r_p_*_0_, Δ*_s_* ≡ (*r_s_*−*r_s_*_0_)/*r_s_*_0_, *r*_p0_ and *r*_s0_ are the complex reflectivities for *p*- and *s*-polarized lights reflected from bare substrates, and *r*_p_ and *r*_s_ are the complex reflectivities for *p*- and *s*-polarized lights reflected from substrates covered by a thin biomolecular layer. Also, *θ* is the incident angle, *ε*_0_, *ε*_s_, and *ε*_d_ are the dielectric constants of the ambient (buffer), the biomolecular layer, and the substrate (glass), respectively. For an Nd-YAG laser with *λ* = 532 nm, *ε*_o_ = 1.788 (1× phosphate buffered saline, PBS), *ε*_s_ = 2.307 (BK7 glass substrate), and *ε*_d_ is real for a protein layer, only the imaginary part of Δ*_p_* − Δ*_s_* is nonzero:

(3) Im{Δp−Δs}≅α(ɛd−ɛ0)(ɛd−ɛs)ɛd(dλ)

Hence by monitoring the measurable quantity Im{Δ*_p_* − Δ*_s_*} over time, the corresponding changes in thickness *d* and/or dielectric constant *ε*_d_ can be determined.

**Figure 1.**
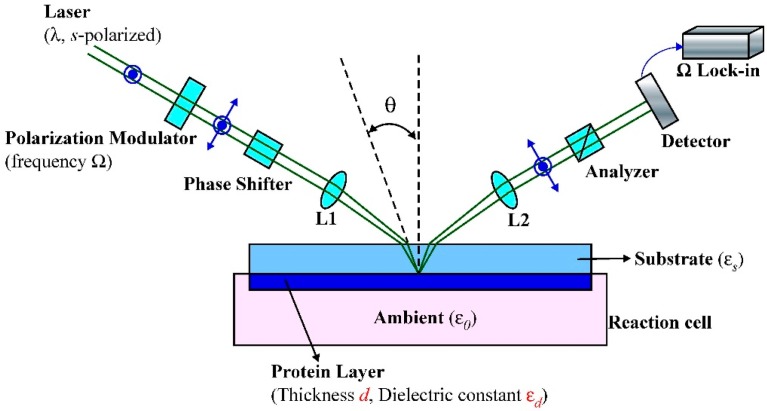
Optical setup of OI-RD. The reaction cell is put above a stirrer. Abbreviations: L1, focal lens; L2, microscope objective.

As illustrated in Figure 1, an *s*-polarized Nd-YAG laser beam passes through a photoelastic modulator (PEM), a phase shifter (PS), and a lens (L1) with *f* = 50 cm focusing onto the biomolecular surface. The phase shifter is a polarizer used to adjust the phase difference between *p*- and *s*-polarized components of the monochromatic light. After reflection, the laser beam passes through a 10× microscope objective (L2, *f* = 20 cm) for separating lights from front-surface and back-surface of the substrate. After an analyzer (A), the intensity of the transmitted beam is detected with a photodiode detector (PD). In the current setup, the back-surface incident angle was θ = 35.7°, giving an α value of 46.17.

### 2.2. Materials and Experimental Conditions

Before reaction, a clean epoxy-coated glass slide (CEL Associates, Pearland, TX, USA) was mounted onto a home-made reaction chamber. Purified Immunoglobulin G from non-immunized rabbit (IgG-RB) and polyclonal antibody purified from goat serum against the F_c_ fragment of rabbit IgG (Goat Anti-RB) were purchased from Jackson ImmunoResearch Laboratories (West Grove, PA, USA). The stock concentrations for IgG-RB and its antibody were 11 mg/mL and 2.4 mg/mL, both in 1× phosphate buffered saline (PBS), respectively. Proteins bind covalently to epoxy groups through reactions via primary amine groups in lysine or arginine residues on their surfaces. Then this chamber was mounted on top of a magnetic stirrer (Corning Incorporated, Nagog Park Acton, MA, USA) which is on during the whole monitoring process. It was shown that under suitable stirring conditions, the binding kinetics is much more reaction-limited than diffusion-limited [29–31]. The experimental processes are as follows: First, 120 mL of 1× PBS (pH 7.4, 0.22 μm filtered) was added into the chamber and the recording started. A small volume of stock IgG-RB was pipetted into the chamber to obtain the desired concentration. After the reaction was complete, the solution was sucked out with a syringe. Then 120 mL of fresh 1× PBS was added again, and finally a small volume of stock Goat Anti-RB was pipetted into the cell. The recording was stopped after the dynamic equilibrium was reached. In biotin-streptavidin-biotinylated bovine serum albumin (BSA) reactions, purified streptavidin was purchased from Sigma-Aldrich (St. Louis, MO, USA) and pre-diluted into 5 mg/mL stock concentration in 1× PBS. Biotinylated BSA was prepared as detailed in the reference [4,32]. The experimental procedure was similar to that of antibody-antigen reactions as described above.

### 2.3. Improvement of OI-RD with Prism and Photodiode Array

**Figure 2.**
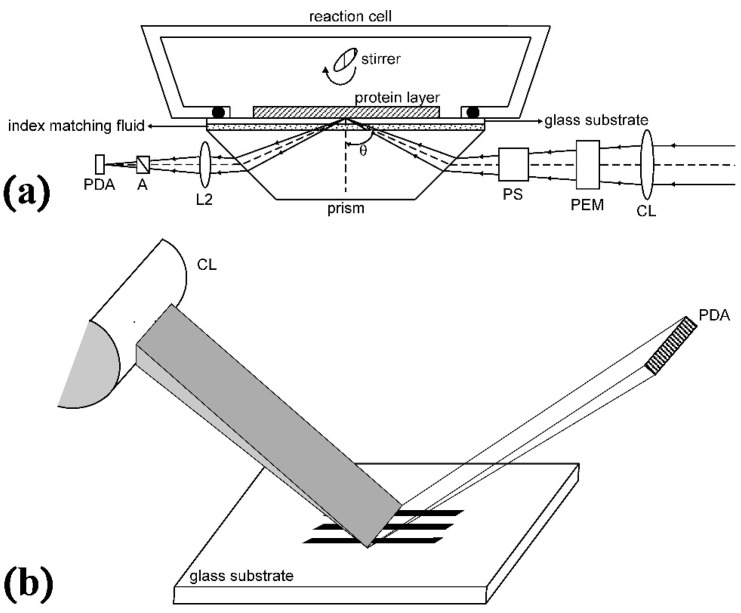
Modified OI-RD setup. (**a**) Top view: L1 and Detector in Figure 1 are replaced with a cylindrical lens (CL) and photodiode array detector (PDA), respectively; (**b**) Side view: After a cylindrical lens, the laser beam is focused into a thin line on the samples.

In order to improve the sensitivity and performance, we have modified the OI-RD microscope with other optical components. First, the total internal reflection (TIR) geometry is incorporated into the system via a prism. Under this TIR condition, the laser beam is totally reflected from the protein-glass interface without being transmitted into the ambient, and almost 100% of the incident power is reflected back to the detector (without TIR, only 0.5% is reflected). Besides, a customized 152-element line photodiode array detector (PDA) and printed line-shaped microarrays (see next section) are used for multi-channel detection. The modified OI-RD setup is shown in Figure 2((a): top view; (b): side view). The focal lens (L1) in Figure 1 is replaced with a cylindrical lens (CL, *f* = 30 cm) which focused the beam into a 12 mm-long thin line on the microarray surface. After reflection, a 10× microscope objective (L2, *f* = 20 cm) is used to project the magnified, inverted line onto the PDA. By scanning all 152 diodes, we can acquire the line profile of all printed samples and bare substrates versus diode number. We then select some diodes representing various proteins and substrates, and monitor their real-time signals simultaneously. The signal drifts over time can therefore be corrected out and compensated by subtracting the background variation of bare substrates. In this setup, the incident angles at the prism and the microarray surface are 31° and 65°, respectively, giving *α* = −14.46.

### 2.4. Printing Microarrays and the Following Reacting Conditions

Microarrays were printed on epoxy-coated glass slides with an OmniGrid 100 capillary contact-printing robot (Genomic Solutions, Ann Arbor, MI, USA). The first batch is a lined-shaped microarray containing BSA, 5× biotinylated BSA, and 40× biotinylated BSA (where 5× and 40× represent the average numbers of biotin molecules bound to one BSA molecule). The second line-shaped microarray contains BSA, 40× biotinylated BSA and two different peptides conjugated BSA (HPYPP-BSA and LHPQF-BSA, both have 3 peptide molecules bound to one BSA in average). Biotinylated BSA and peptide conjugated BSA were prepared as described reference [4,32]. All samples in these two microarrays were printed at a concentration of 0.5 mg/mL in 1× PBS. Before further processing, the printed slides were stored in slide boxes for at least 24 h until the printed spots were completely dried. Before reaction, the slide was mounted against the prism onto the reaction chamber. An index matching fluid with a refractive index of 1.52 (purchased from Cargille Labs, Cedar Grove, NJ, USA) was pipetted between the slide and the prism to ensure TIR occurring at the microarray surface. Then 180 mL of 1× PBS was added into the chamber, and the recording started after selecting desired diodes. In the first 20 min of stirring, the slide was washed to remove excess unbound proteins and buffer precipitates. Then 20 mL of 5 mg/mL BSA in 1× PBS was added for blocking. After another 10 min, the solution was sucked out with a syringe, and 120 mL of fresh 1× PBS was added again. From our experimental results (data not shown), this blocking condition (0.5 mg/mL BSA for 10 min) is appropriate and enough for effectively quenching unreacted epoxy groups. Finally, a small volume of stock reagent was pipetted into the chamber to obtain the desired concentration, and the recording stopped after the dynamic equilibrium is reached.

## 3. Results and Discussion

### 3.1. OI-RD Detection of Antibody-Antigen Capture

The kinetics of antigens (or antibodies) binding to solid substrates has been an important area in immunodiagnostics for many years [33]. The following reactions with their corresponding antibodies (or antigens) have also been widely studied, simply because these antibody-antigen reactions provide very good platforms for kinetic demonstrations [34–36]. In this study, we used immunoglobulin G from non-immunized rabbit (IgG-RB) as the antigen, and its polyclonal antibody (Goat Anti-RB, purified from goat serum) is supposed to bind against its F_c_ fragment. In OI-RD measurements, the surface mass density (*Γ*) and surface number density (*σ*) of antigens or antibodies (molecular weight = 150 kDa) can be expressed in terms of Im{Δ*_p_* − Δ*_s_*} as *Γ* = (212.3 ng/mm^2^) and *σ* = (8.52 × 10^11^ /mm^2^) × Im{Δ*_p_* − Δ*_s_*} [23,32].

**Figure 3.**
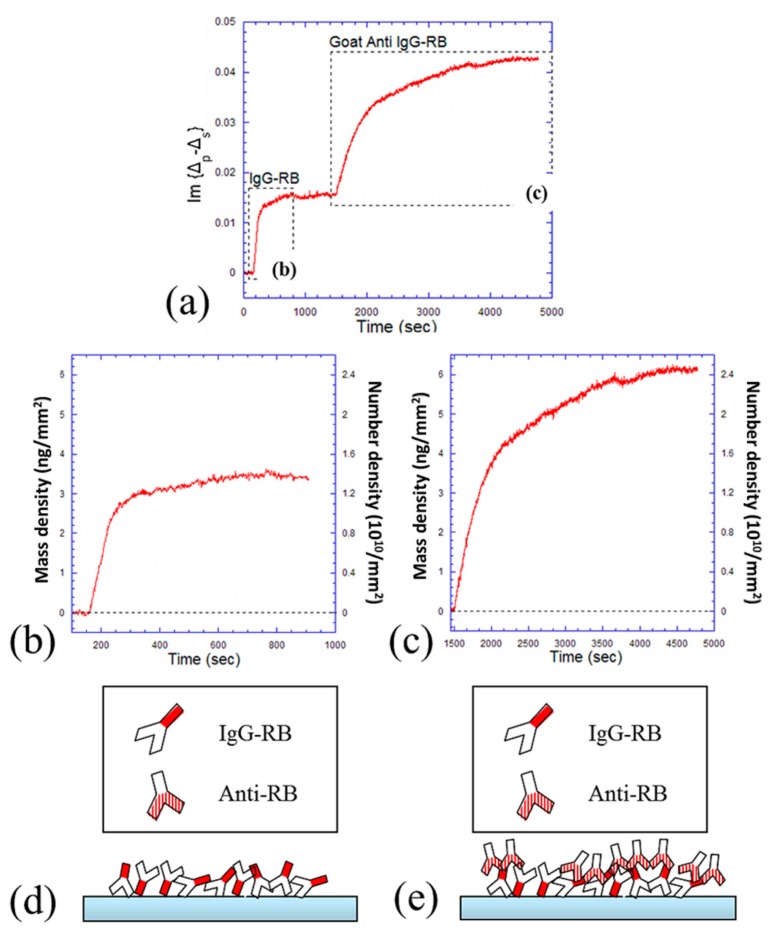
Kinetics of antibody-antigen capture. (**a**) OI-RD detection of antigen (67 nM of IgG-RB) binding to epoxy surface and following antibody (33 nM of Goat Anti-RB) binding to antigen; (**b**) Kinetic curve of IgG-RB binding to epoxy surface; (**c**) Kinetic curve of Goat Anti-RB binding to IgG-RB; (**d**) Cartoon depiction of process (b); (**e**) Cartoon depiction of process (c).

Figure 3a shows the kinetic curve of IgG-RB (67 nM) binding onto epoxy-coated substrate and the following Goat Anti-RB (33 nM) binding to IgG-RB in a unit of Im{Δ*_p_* − Δ*_s_*}. The dynamic equilibrium for IgG-RB immobilizing onto epoxy surface is reached in less than 10 min. However, it takes much longer (about an hour) for Goat Anti-RB to bind to its partner IgG-RB. This is because antibodies are large molecules, and it takes much time for them to adjust themselves, find the binding sites (*i.e.*, the epitopes on antigens), and then settle down. These two processes are shown separately in Figure 3b,c, in units of surface mass density and surface number density. Using the ellipsoid model [23,32], a human IgG molecule has a size of 4.4 nm × 4.4 nm × 23.5 nm [37]. The calculated surface mass densities are 2.5 ng/mm^2^ in a “side-on” IgG monolayer and 13 ng/mm^2^ in an “end-on” IgG monolayer [23]. In OI-RD measurements, the equilibrium mass density of IgG-RB immobilized onto epoxy surface is about 3.4 ng/mm^2^. This indicates that approximately a “side-on” IgG-RB monolayer is formed on the surface. The following mass density change of Goat Anti-RB is about 6.1 ng/mm^2^, and this can be explained as the accumulation of mixed “side-on” and “end-on” Goat Anti-RB molecules. In the number density point of view, nearly two antibodies are bound to one antigen. This is because there are many different epitopes on the F_c_ fragment of one antigen, where more than one polyclonal antibody can bind to through their two F_ab_ fragments. These two processes are also cartooned in Figure 3d,e.

**Figure 4.**
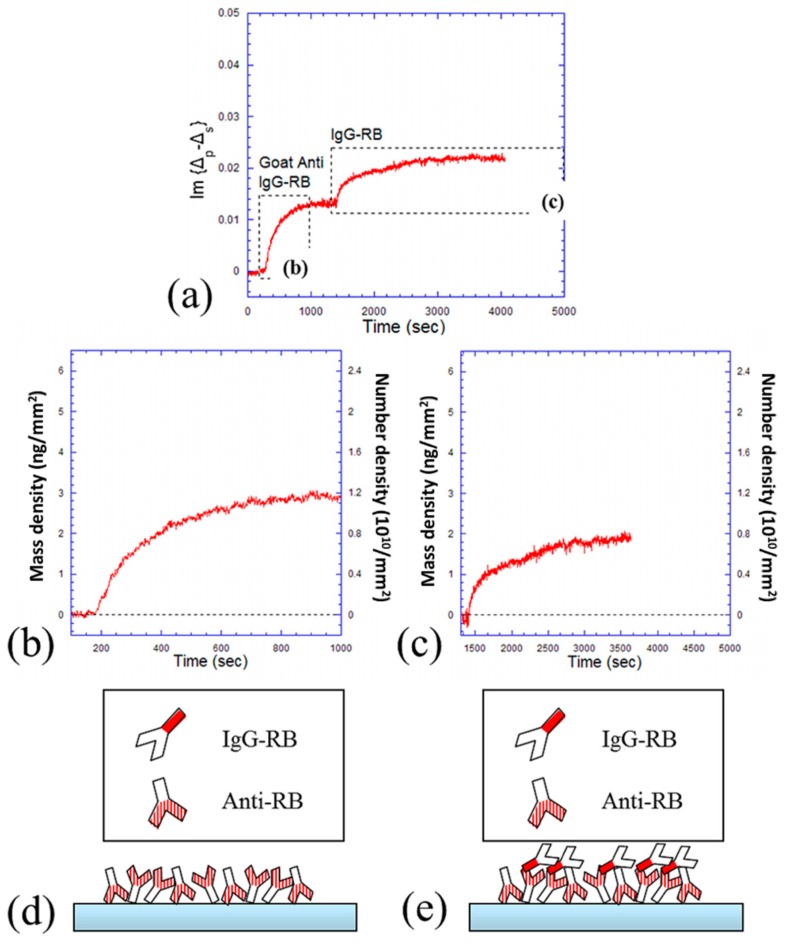
Kinetics of antigen-antibody capture. (**a**) OI-RD detection of antibody (33 nM of Goat Anti-RB) binding to epoxy surface and following antigen (67 nM of IgG-RB) binding to antibody; (**b**) Kinetic curve of Goat Anti-RB binding to epoxy surface; (**c**) Kinetic curve of IgG-RB binding to Goat Anti-RB; (**d**) Cartoon depiction of process (b); (**e**) Cartoon depiction of process (c).

Next, a reverse process was done, where Goat Anti-RB (33 nM) was first reacted with the epoxy surface and then followed by IgG-RB (67 nM) reactions. The kinetic curve is shown in Figure 4a in a unit of Im{Δ*_p_* − Δ*_s_*}. It is shown that the time for Goat Anti-RB binding to epoxy surface is shorter than that for the following IgG-RB binding to its partner (about 15 min and 35 min, respectively). These two processes are shown separately in Figure 4b,c. The equilibrium mass density of immobilized Goat Anti-RB is about 2.9 ng/mm^2^, corresponding to a “side-on” monolayer on the surface. But the following equilibrium value is much smaller (only about 1.9 ng/mm^2^), which means that not even a monolayer of IgG-RB bound to its antibody. The corresponding number density indicates that only about two antigens bound to three antibodies in average. These two processes are also cartooned in Figure 4d,e. There are two possible reasons to explain the discrepancy between antibody binding to surface antigen and antigen binding to surface antibody. First, since all immobilized antibodies are randomly orientated on the surface, some of their F_ab_ fragments might be hidden and hence not available for reactions. Second, there are many different epitopes on the F_c_ fragment of one antigen (and therefore the surface area for one epitope is small), so it is difficult for the antigen to adjust itself and find the correct epitope for binding to its partner.

### 3.2. OI-RD Detection of Protein Binding to Small-Molecule Ligands

**Figure 5.**
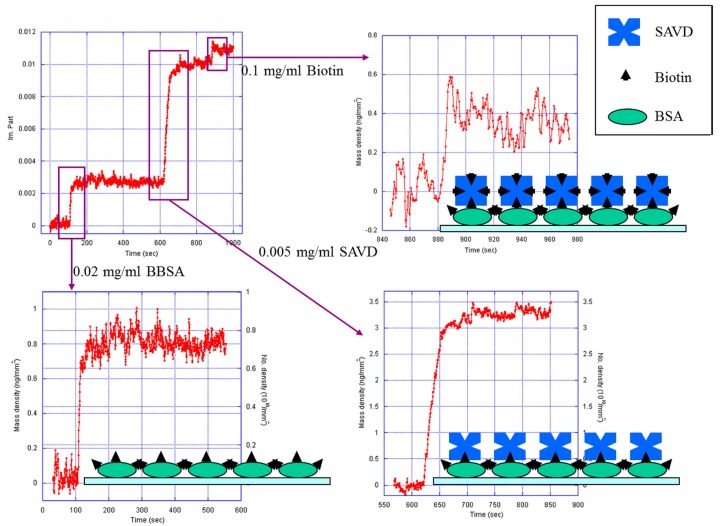
Kinetics of streptavidin binding to biotinylated BSA: Kinetic curve of biotinylated BSA (0.3 μM) binding to epoxy surface, streptavidin (83 nM) binding to biotin, and biotin (0.1 mg/mL) binding to streptavidin. Cartoon depictions of all processes are also shown.

In addition to protein-protein interactions, OI-RD microscopy can be applied to detect protein-small molecule interactions. Streptavidin is a tetrameric protein with a molecular weight of 60 kDa, and each of its subunits has a single binding site for biotin with an affinity constant of 10^14^ ∼ 10^15^ M^−1^ in bulk phase. The surface mass and number densities of bound streptavidin molecules are calculated as *Γ* = (212.3 ng/mm^2^) × Im{Δ*_p_* − Δ*_s_*} and *σ* = (2.13 × 10^12^ /mm^2^) × Im{Δ*_p_* − Δ*_s_*}, respectively [32]. The kinetic curve of biotinylated BSA (0.3 μM) binding onto epoxy surface, streptavidin (83 nM) reacting with surface biotin, and the following biotin (0.1 mg/mL)-streptavidin reaction is shown in Figure 5. Individual processes together with cartoon depictions are also shown. The first step is similar to immobilizing BSA molecules onto epoxy surface, since biotin molecules are very small comparing with BSA scaffolds. In equilibrium, the mass density is only about 0.64 ng/mm^2^, indicating that less than a “side-on” monolayer is formed. Streptavidin is a roughly spherical molecule with a diameter of 5 nm [38,39], giving a monolayer mass density of 4.0 ng/mm^2^. Our measured signal gives a 1.49 ng/mm^2^ mass density of bound streptavidin, which is less than a monolayer simply because the restriction in the number of pre-immobilized biotinylated BSA molecules. Biotin is a small molecule with a molecular weight of about 244 Da. Successful observation of biotin binding to surface streptavidin implies that this OI-RD technique is applicable in detecting small molecule compounds with molecular weights around 100 Da.

### 3.3. Use OI-RD for Multi-Channel Detection

We have modified the OI-RD microscope with a prism and a photodiode array (PDA) detector to enable high-sensitivity and multi-channel detection in line-shaped microarrays. Usually one diode is selected for one particular protein or substrate, and all these selected diodes are monitored simultaneously. Each binding curve is compensated by subtracting out its nearest substrate signal to get rid of noise drift. In this setup, *α* equals to −14.46, and the surface mass density can be expressed as *Γ* = (677.9 ng/mm^2^)× Im{Δ*_p_* − Δ*_s_*}.

**Figure 6.**
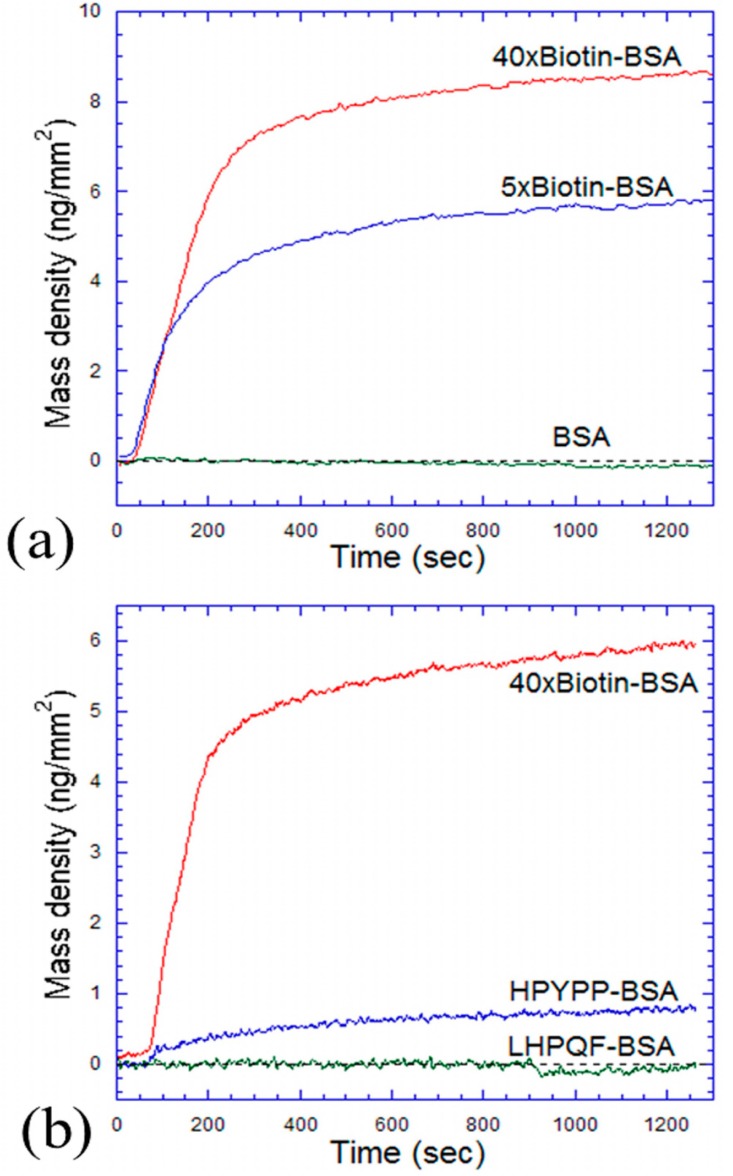
(**a**) OI-RD detection of streptavidin reacting with a line-shaped microarray containing BSA, 5× and 40× biotinylated BSA. 83 nM of streptavidin was added; (**b**) OI-RD detection of avidin reacting with a line-shaped microarray containing 40× biotinylated BSA, HPYPP and LHPQF conjugated BSA. 83 nM of avidin was added. All curves in (a) and (b) were compensated by subtracting the substrate signal.

The binding curves of streptavidin reacting with BSA, 5× biotinylated BSA, and 40× biotinylated BSA are shown in Figure 6a. This microarray slide was washed, blocked, and then reacted with streptavidin at 83 nM. The observed reaction rates are almost the same for both 5× and 40× biotinylated BSAs, and it takes about 10 min for both reactions to reach equilibrium. The equilibrium mass densities of bound streptavidin molecules for 5× and 40× biotinylated BSAs are 5.7 ng/mm^2^ and 8.5 ng/mm^2^, respectively, and these values are much higher than that of a streptavidin monolayer. Because there are many biotin molecules available per BSA, they can stick out (through linkers) and provide more binding sites for streptavidin molecules. Here, BSA is a negative control because it gives no signal change. Another similar experiment was done and shown in Figure 6b. 40× biotinylated BSA (used as a positive control), HPYPP conjugated BSA, and LHPQF conjugated BSA (used as a negative control) were reacted with avidin at 83 nM. It is observed that avidin reacted with both 40× biotinylated BSA (with an equilibrium mass density of 5.7 ng/mm^2^) and HPYPP conjugated BSA (with an equilibrium mass density of 0.8 ng/mm^2^), but not LHPQF conjugated BSA.

## 4. Conclusions

One of the most important issues in proteomics is to be able to observe, measure, and analyze biomolecular interactions in a quantitative manner. Although fluorescence is very widely used, those extrinsic tags may change the conformational and functional properties of protein molecules. The oblique-incidence reflectivity difference (OI-RD) technique is applicable in label-free kinetic measurements of protein-biomolecule interactions. In this article, we demonstrated using the OI-RD microscope to monitor the kinetics of protein binding to epoxy functionalized substrates, antibody-antigen bindings, and protein reacting with small-molecule ligands. With minor modifications, we are able to increase the sensitivity of OI-RD to a comparable value with other label-free methods such as SPR and RIfS. Additionally, multi-channel detection eliminates signal drift and also enables simultaneous measurements. We believe that this OI-RD technique can be applied in high-throughput screening, both real-time and end-point, of protein-binding ligands, an important process in discovering new drugs.
